# In vitro and in silico validation of *CA3* and *FHL1* downregulation in oral cancer

**DOI:** 10.1186/s12885-018-4077-3

**Published:** 2018-02-17

**Authors:** Cláudia Maria Pereira, Ana Carolina de Carvalho, Felipe Rodrigues da Silva, Matias Eliseo Melendez, Roberta Cardim Lessa, Valéria Cristina C. Andrade, Luiz Paulo Kowalski, André L. Vettore, André Lopes Carvalho

**Affiliations:** 1 Department of Head and Neck Surgery, A. C. Camargo Cancer Hospital, São Paulo, Brazil; 2 Laboratory of Cancer Genetics, Ludwig Institute for Cancer Research, Sao Paulo, Branch Brazil; 3 Molecular Oncology Research Center, Barretos Cancer Hospital, Barretos, Brazil; 4 Embrapa Informatica Agropecuaria, Campinas, Brazil; 5 Discipline of Hematology and Hemotherapy, Universidade Federal de São Paulo, UNIFESP, São Paulo, Brazil; 6 Department of Science Biology, Universidade Federal de São Paulo, UNIFESP, Diadema, Brazil; 7 Department of Head and Neck Surgery, Barretos Cancer Hospital, Barretos, São Paulo Brazil

**Keywords:** OSCC, Gene expression, Methylation, *CA3*, *FHL1*

## Abstract

**Background:**

Aberrant methylation is a frequent event in oral cancer.

**Methods:**

In order to better characterize these alterations, a search for genes downregulated by aberrant methylation in oral squamous cell carcinoma (OSCC) was conducted through the mining of ORESTES dataset. Findings were further validated in OSCC cell lines and patients’ samples and confirmed using TCGA data. Differentially expressed genes were identified in ORESTES libraries and validated in vitro using RT-PCR in HNSCC cell-lines and OSCC tumor samples. Further confirmation of these results was performed using mRNA expression and methylation data from The Cancer Genome Atlas (TCGA) data.

**Results:**

From the set of genes selected for validation, *CA3* and *FHL1* were downregulated in 60% (12/20) and 75% (15/20) of OSCC samples, respectively, and in HNSCC cell lines. The treatment of cell lines JHU-13 and FaDu with the demethylating agent 5'-aza-dC was efficient in restoring *CA3* and *FHL1* expression. TCGA expression and methylation data on OSCC confirms the downregulation of these genes in OSCC samples and also suggests that expression of *CA3* and *FHL1* is probably regulated by methylation. The downregulation of *CA3* and *FHL1* observed in silico was validated in HNSCC cell lines and OSCC samples, showing the feasibility of integrating different datasets to select differentially expressed genes in silico.

**Conclusions:**

These results showed that the downregulation of *CA3* and *FHL1* data observed in the ORESTES libraries was validated in HNSCC cell lines and OSCC samples and in a large cohort of samples from the TCGA database. Moreover, it suggests that expression of *CA3* and *FHL1* could probably be regulated by methylation having an important role the oral carcinogenesis.

**Electronic supplementary material:**

The online version of this article (10.1186/s12885-018-4077-3) contains supplementary material, which is available to authorized users.

## Background

Squamous cell carcinoma (SCC) is the most frequent histological subtype of oral cavity cancers. This disease originates from the epithelial tissue that covers the entire aero digestive tract and accounts for more than 90% of all malignancies in that anatomical region [1]. This cancer site is among the most common worldwide and a major cause of morbidity and mortality [2]. Despite extensive research and improvements in diagnostic methods and treatment approaches, the five-year overall survival rate for oral squamous cell carcinoma (OSCC) patients have only improved marginally. Investigation of molecular targets and signaling pathways to design appropriate therapeutic, follow-up and monitoring strategies may have the potential to improve survival [3].

Several studies in oral carcinogenesis point to an important relationship between aberrant DNA methylation at the promoter of tumor suppressor genes and their inactivation [4–9]. DNA methylation is a frequent epigenetic event that occurs by the addition of a methyl group (-CH_3_) to a cytosine (C) situated at a 5′ position of a guanine (G) in CpG dinucleotides of superior eukaryotic cells [10, 11]. Genetic and epigenetic events can confer competitive advantages to a cell leading to a cancer phenotype [12, 13], therefore a wide transcriptome analysis revealing the molecular mechanisms underlying cancer environment is important [14, 15].

The integration of different data sets such as serial analysis of gene expression (SAGE), expressed sequence tags (ESTs) and open reading expressed sequence tags (ORESTES), provide powerful platforms to evaluate gene expression data in cancer tissues [16]. The ORESTES data set was developed by a Brazilian research group during the Human Cancer Genome Project, yielding more than 1 million sequences representing parts of mRNAs expressed in different tumors [17]. This technology allowed the acquisition of sequences from the central codifying region of transcripts by using random primers [18] and was used to identify differentially expressed genes and several transcriptomes [15, 17, 19–21]. All sequences produced in these projects are available in public databases.

The Cancer Genome Atlas (TCGA) Research Network is a multi-institutional consortium focused on the comprehensive clinical and molecular profiling of 32 different tumor types [22]. Head and neck squamous cell carcinoma (HNSCC) sample collection from TCGA data portal contains 528 cases, including samples from oral cavity, larynx, tonsils, base of tongue, pharynx and lips [22].

The use of gene expression based molecular markers as tools to improve the understanding of the biological mechanisms involved in oral cancer carcinogenesis opens the potential for the discovery of new therapy targets, better prediction of patient outcome, therapy choice and surveillance strategies, improving patient quality of life and survival rates. Thus, in this study, we used bioinformatics analysis from head and neck ORESTES libraries to identify differentially expressed genes in oral cancer and to investigate whether gene downregulation was a consequence of aberrant methylation. To validate the findings, we performed the pharmacological unmasking of OSCC cell lines through their treatment with a demethylating agent, and analyzed the gene-expression level of selected genes in patients´ samples. We further confirmed the results by analyzing methylation and RNA expression data from the TCGA database.

## Methods

### In silico analysis of ORESTES data

Downregulated transcripts were selected from ORESTES data available at the National Center for Biotechnology Information (NCBI) database. A bioinformatic analysis generated a list of differentially expressed genes in different head and neck squamous cell carcinoma subsites in comparison to their correspondent normal tissue. The program BlastN was used to compare the 946,260 ORESTES sequences deposited at NCBI with the 29,529 reference sequences of human genes presented at the RefSeq database [23]. The best hit of an ORESTES sequence with a human gene was selected to define from which gene this sequence was generated, with no visual inspection. Only hits with *e-values* better than 1 × 10^− 10^ were considered, thus, 570,214 ORESTES were included in this analysis. Results were then loaded into a relational database.

Only normal and tumor head and neck ORESTES libraries were analyzed (see Additional file 1: Table S1) which were compared by three ways: (1) normal larynx libraries plus normal hypopharynx libraries were compared with oral cavity tumor library; (2) normal larynx libraries were compared with larynx tumor libraries; and (3) normal hypopharynx libraries were compared with hypopharynx tumor libraries. The Fisher Exact Test was applied to identify genes differentially expressed and a *p*-value < 0.05 was used to consider statistical significance.

### Downregulated candidate genes selection

By using available web tools (see Additional file 2: Table S2), several criteria were applied to define the best downregulated candidate genes such as: (1) the presence of CpG island in the promoter region; (2) ESTs expression evaluation in head and neck tissue; and (3) data from a literature review. In this last criterion, genes with biologic functions related to carcinogenesis and those described as downregulated in other tumors were included, while genes previously described as oncogenes or overexpressed were excluded.

### OSCC specimen and control samples

Twenty primary OSCC specimens from patients surgically treated at the Department of Head and Neck Surgery, A. C. Camargo Hospital and available at the Tumor Bank of this institution were included. All tissues were subjected to intraoperative frozen section evaluation to select necrosis and calcification-free areas and immediately stored at − 80°C until nucleic acid extraction. Ten histologically normal oral mucosa samples were collected from healthy donors undergoing dental and pre-prosthetic surgeries and were used as control tissue. Written informed consent was obtained from all OSCC patients and healthy donors at the time of enrollment and all aspects of this investigation were approved by the Ethics Committees of A. C. Camargo Hospital (process number 737/05).

### Tumor cell lines

HNSCC cell lines JHU-12, JHU-13, JHU-19, JHU-28 were kindly provided by Dr. Joseph Califano (Department of Otolaryngology and Head and Neck Surgery - Jonhs Hopkins University). FaDu cell line was acquired from ATCC (American Type Cell Collection – Rockville, MD). JHU-12, JHU-13, JHU-19, JHU-28 cell lines were maintained in RPMI medium and FaDu in MEM medium, supplemented with 10% fetal bovine serum in the presence of antibiotics at 37 °C with 5% CO_2_.

### 5′-aza-2′-deoxycytidine treatment

To investigate a possible role of epigenetic in the downregulation of selected genes, 10^5^ JHU-13 and FaDu cells were seeded on day 0 and treated with 1 μM of the demethylating agent 5-aza-dC (Sigma-Aldrich, St. Louis, MO) for 3, 5 and 7 days. DNA and RNA were extracted at days 0, 3, 5 and 7 and stored at − 80°C. The level of gene expression of the genes selected was tested before and after treatment with the demethylating agent, following the procedures described next.

### RNA extraction and cDNA synthesis

Total RNA from normal and tumor samples was extracted using the TRIzol Reagent (Invitrogen, Carlsbad, CA, USA) according to the manufacturer’s protocol. Total RNA from HNSCC cell lines was extracted by cesium chloride gradient ultracentrifugation method. Briefly, cells were homogenized in 9 mL of lyses solution (4 M guanidinium isothiocyanate, 2 mM sodium citrate pH 7.0; 0.1 M β-mercaptoethanol). The cell lysate was then transferred to an ultracentrifuge tube with 4 mL of cesium chloride solution (5.7 M CsCl; 1 M sodium acetate) and submitted to 29,000 rpm for 20 h at 20°C. Following centrifugation, the RNA pellet was dissolved in 100 μL of RNAse-free water. All extracted RNA samples were quantified in the spectrophotometer NanoDrop-ND 1000 (Thermo Scientific, Wilmington, DE) and analyzed by electrophoresis in 1% agarose gel stained by 0.5 μg/mL ethidium bromide.

Two micrograms of template RNA were used for first-strand cDNA synthesis using oligo (dT) primers and the reverse transcriptase Superscript III (Invitrogen, Carlsbad, CA) following manufacturer’s instructions. The cDNA product was diluted 10 times prior to use. Quality cDNA control was performed by the amplification of an *ACTB* (NM 001101) fragment using forward (5´-CACTGTGTTG GCGTACAGGT-3′ and reverse primers (5´-TCATCACCATTGGCAATGAG-3′). Reactions were carried out under the following conditions: 94 °C for 2 min, followed by 35 cycles at 94 °C for 30 s, 58 °C for 45 s, 72 °C for 45 s and 72 °C for 7 min. PCR products were evaluated by electrophoresis in 1% agarose gel stained with 0.5 μg/mL ethidium bromide.

### Validation of mRNA expression changes in HNSCC cell lines by RT-PCR

The expression level of ten genes *(CA3, FHL1, HMGN4, FSTL1, NFE2L1, SAR1B, C9orf64, ANXA6, WDR26, CCN1*) was evaluated by Reverse Transcription PCR (RT-PCR) in five HNSCC cell lines. Primer sequences, amplicon sizes, MgCl_2_ concentration and annealing temperatures are available in Table 1.

**Table 1:** Primer sequences, product size, MgCl_2_ concentration and annealing temperatures used in RT-PCR analyses

Gene	Primer sequence (5′-3′)	Product size (bp)	MgCl_2_ (mM)	Annealing temperature (°C)
*CA3*	F: TGAAGCAGCGCGATGGGAT R: GTCAGAGCTCACGGTCATGGGC	260	2	66
*FHL1*	F: CCGCTTCTGGCATGACACCT R: ACGGTCCCCTTGTACTCCACG	189	2	66
*ANXA6*	F: -CCGGCACAGATGAAAAGGCTC R: TTCTCCTCCCTCCTCACGATGC	191	2	66
*WDR26*	F: TGCCAATTGCGGAGCTGACA R: CGTCTGCTCCAAATTCACCATCAA	196	2	66
*HMGN4*	F: CCTTCCCTCGCCTTCCTGTTCC R: TGTCCTCCTCACGCTGTTCCTGG	182	1	66
*C9orf64*	F: AGGCTCTTTTCTCAACTGCGTCCGT R: AGCAGCCATCTCCTTTTCCTTCCA	191	2	66
*FSTL1*	F: CCCAGACCCAGACAGAGGAGGAG R: ACTGGTGATTTGGCGACTGTAGCA	203	2	66
*CCN1*	F: GCAATTCAGAGGATCCATG R: GGTGTGCTTGAGGGGACGGTAG	220	3	55
*SAR1B*	F: ACCACGAAAGGCTGTTAGAGTCAAAA R: AACCAAACATCTCTCGCAACCTCTC	146	2	66
*NFE2L1*	F: ACGGAACCTGCTAGTGGATGGAGA R: CTGTTATGCTGGAAATGTCTGCTGGA	167	1	70

### Real-time quantitative RT-PCR (qRT-PCR) analysis

To validate the expression profile data from HNSCC cell lines in clinical samples, mRNA levels of the selected candidate genes *CA3 and FHL1* were tested by qRT-PCR on 20 OSCC cases and 10 normal oral samples. All qRT-PCR analyses were performed on an ABI 7000 Sequence Detection System (Applied Biosystems, Foster City, CA) using SYBR Green (Applied Biosystems, Faster City, CA) for detection. Tests for optimal annealing conditions, as well as melting curve analysis to confirm amplification specificity were conducted for each set of gene-specific primers.

The amplification reactions were carried out using 2 μL of cDNA template in a final volume of 20 μL containing: 1 U of Platinum Taq DNA Polymerase (Invitrogen, Grand Island, NY), 1X polymerase buffer, 2 mM MgCl_2_, 200 μM of each dNTP, 20 pmol of each primer, 5% DMSO and 0.2 μL of SYBR Green I (working dilution 1:100; Applied Biosystems, Faster City, CA). The standard amplification protocol consisted of an initial denaturation step for 2 min at 95 °C, followed by 40 amplification cycles at 95 °C for 15 s, annealing at 68 °C (*CA3*) or 72 °C (*FHL1*) for 30 s and extension at 72°C for 30 s.

Experiments were performed in triplicates and mean values were used for gene expression calculations. The relative gene expression level was estimated using the 2^-ΔΔCt^ method [24]. Each sample data was normalized on the basis of the expression of three reference genes *RPLO*, *PPIA* and *TBP* [21]. The results were expressed as n-fold differences in the relative expression of the reference genes in tumor and the normal samples. A gene was considered downregulated when the expression level was below the arbitrary cut-off adopted (2-fold change downregulation).

### In silico TCGA data analysis

We decided to further validate the results from the selected genes by analyzing the TCGA data on gene expression and methylation available for HNSCC (UNC_IlluminaHiSeq_RNASeqV2 for RNA sequencing data; JHU-USC_HumanMethylation450, for DNA methylation data; and Biotab for clinical data). The data from 14 normal and 312 OSCC samples were all obtained from the TCGA data portal (http://www.cbioportal.org/study?id=hnsc_tcga#summary). Samples included are described in Additional file 3: Table S3. Methylation data for both genes analyzed were targeted by multiple probes, but only mean β-values for each gene were used in statistical analysis. Expression and methylation differences between tumor and normal OSCC samples were tested with independent t-test at 5% significance level. Pearson’s correlation test was performed for *CA3* and *FHL1* mRNA expression and methylation, at 5% significance level. For the *heatmap* graphical representations, *CA3* and *FHL1* mRNA expression levels were dichotomized at 250 and 2300 (normalized counts), respectively. These values were chosen arbitrarily in order to best maximize the capacity of distinction between OSCC and healthy subjects, based in the box-plot graphs presented in Fig. 4. Statistical analyses were performed in SPSS v19. Graphical heatmap representations were constructed with *heatmap3* package of R statistical software [25, 26].

## Results

### Selection of downregulated genes in HNSCC

The program Blastn was run for 946,260 ORESTES against the RefSeq database of human genes, resulting in 570,214 ORESTES selected in this analysis. Comparisons of normal and tumor head and neck ORESTES libraries using Fisher’s Exact Test generated a list with 75 differentially expressed genes (64 downregulated and 11 upregulated genes). The 64 downregulated genes are listed in Additional file 4: Table S4 and the accession numbers for the libraries used are listed in Additional file 5: Table S5. Thirty of these candidates presented CpG islands at their promoter sites and their expression in head and neck was validated by using the Virtual Northern tool from SAGE Anatomic Viewer - Cancer Genome Anatomy Project (SAV-CGAP). This analysis confirmed 24 candidates as downregulated in HNSCC. After a review of the literature data, genes with biologic functions related to carcinogenesis or described as downregulated in other tumors were selected. By the end, we were able to select 10 genes for the assessment of gene expression in HNSCC cell lines (*CA3, FHL1, ANXA6, WDR26, HMGN4, C9orf64, FSTL1, CCN1, NFE2L1* and *SAR1B*).

### Evaluation of selected genes expression in head and neck cell lines

**Fig. 1:**
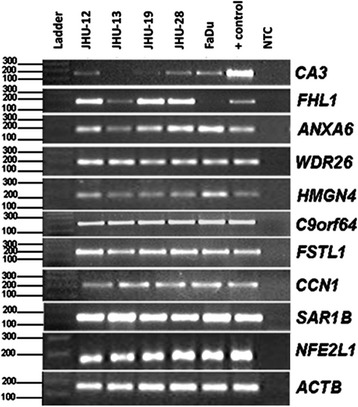
RT-PCR analysis of *CA3, FHL1, ANXA6, WDR26, HMGN4, C9orf64, FSTL1, CCN1, SAR1B* and *NFE2L1* expression in five HNSCC cell lines (represented above). Note, the expression of *CA3* is not detectable in JHU-13 (O13) cell line and *FHL1* is downregulated in FaDu cell line. Legend: (Ladder) 100 bp DNA Ladder (Invitrogen). + positive control (SW 480 tumor cell line) and NTC (no template control). *ACTB* mRNA was used to evaluate quantity in each RT-PCR reaction

Due to the scarcity of RNA obtained from many samples evaluated in the following steps and the high number of genes selected, it would be virtually impossible to evaluate all possible candidate-genes in all samples. Therefore, we performed a first assessment of the expression of these 10 selected genes in cell lines and picked up only the most promising candidates to be evaluated in further experiments with patients’ samples. The results showed that *CA3* was expressed in four of the cell lines evaluated, whereas no mRNA was detected in the JHU-13 cell line, suggesting that this gene is downregulated in this cell line. *FHL1* showed reduced mRNA expression only in FaDu cell line, being expressed in the other cell lines evaluated. The eight remaining genes (*ANXA6, WDR26, HMGN4, C9orf64, FSTL1, CCN1, SAR1B and NFE2L1)* were expressed in all five HNSCC cell lines evaluated (Fig. 1).

### Validation of mRNA expression in OSCC samples by qRT-PCR

**Fig. 2:**
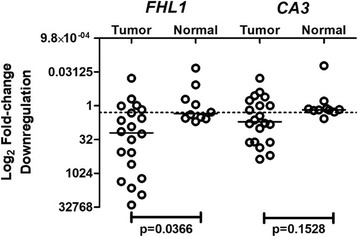
Gene expression profile of *FHL1* and *CA3* in 20 OSCC samples and 10 histologically normal oral mucosa samples. The Y-axis shows the log2 fold-change downregulation of the relative expression (2^-ΔΔCt^). The dotted line indicates the cut-off adopted (2-fold downregulation)

After observing the *CA3* and *FHL1* downregulation in HNSCC cell lines, we sought to test the expression level of these genes in 20 OSCC samples. Clinical and pathological data of the 20 OSCC patients enrolled in this study are as follows: the mean age was 59.4 years, 80% of the patients were male, 70% were tobacco users, 60% had advanced stage tumors (II-IV) and 75% of the tumors were in the oral tongue followed by 20% in the floor of the mouth and 5% in the alveolar ridge. Seventy-five percent (15/20) of the samples showed downregulation of *FHL1* while *CA3* was downregulated in 60% (12/20) of the samples (Fig. 2). The Mann-Whitney test was performed to assess the difference between the expression levels of these two genes between OSCC and normal samples. Results showed a statistically significant difference between these two groups for FHL1 (*p* = 0.0366), but not for CA3 (*p* = 0.1528).

### In silico TCGA validation

**Fig. 3:**
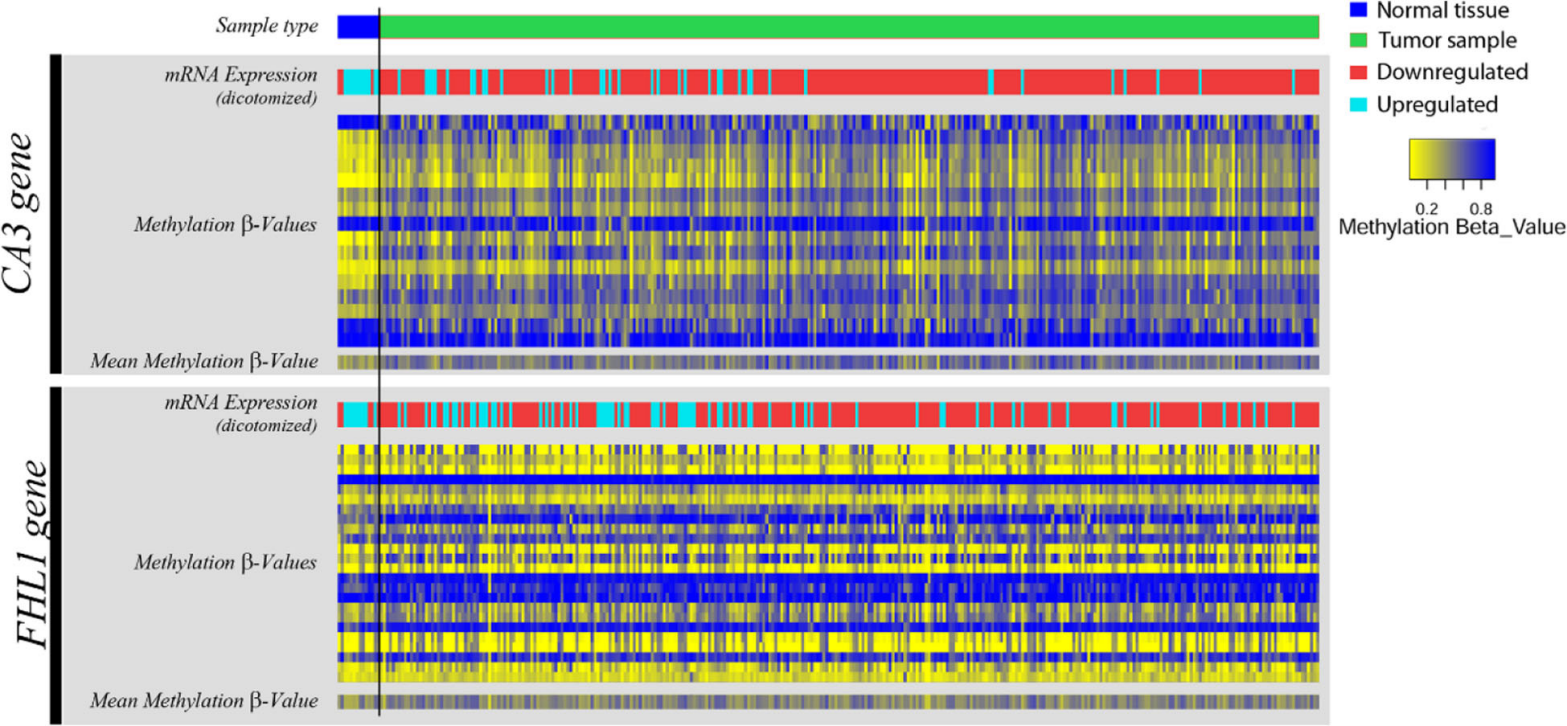
In silico TCGA validation of mRNA expression and methylation data. Heatmap analysis from TCGA data for methylation status (β-values) and mRNA expression of *CA3* and *FHL1* genes in normal and OSCC samples. The black vertical line divides normal from tumor samples

**Fig. 4:**
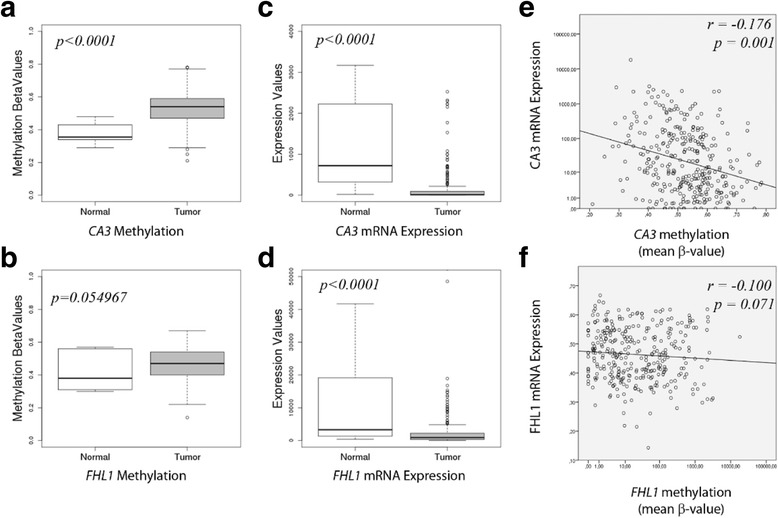
In silico TCGA evaluation of methylation status and mRNA expression of *CA3* and *FHL1*. **a** and **b** boxplots show mean methylation β-values in normal and tumor tissues, for *CA3* and *FHL1* genes, respectively. **c** and **d** boxplots show mRNA expression distributions, in normal and tumor tissues, for *CA3* and *FHL1* genes, respectively. Statistical *p* values denote Student’s *t*test between normal and OSCC samples. **e** and **f** show Pearson’s correlation of mean methylation β-values and mRNA expression, for *CA3* and *FHL1* genes, respectively

To validate the results obtained with the 20 OSCC patient samples and cell lines in a larger cohort, we analyzed publicly available TCGA data of DNA methylation and mRNA expression (Figs. 3 and 4). Supervisionized heatmap (by sample type) of *CA3* methylation β-values showed a clear separation in samples, where normal samples were frequently hypomethylated for most of the probes (Fig. 3). *FHL1* methylation heatmap did not show a clear separation (Fig. 3). Statistical analysis of mean methylation β-values supports these observations, for *CA3* and *FHL1* (*p* < 0.0001 and *p* = 0.055, respectively; Fig. 4a and c). In addition, mRNA expression values also showed a clear discrimination of normal and tumoral samples, where most of the tumor samples presented downregulation of both *CA3* and *FHL1* genes (*p* < 0.0001 – Fig. 4b and d). Pearson’s correlation analysis showed a significant correlation between mRNA expression and mean methylation for the *CA3* gene (*r* = − 0.176; *p* = 0.001 – Fig. 4b) and a trend on this correlation for *FHL1* (*r* = − 0.100; *p* = 0.071 – Fig. 4d). Although statistical analysis of *FHL1* methylation was not significant, all results toghether suggest that expression of *CA3* and *FHL1* is probably regulated by methylation.

### Expression evaluation after 5′-aza-dC treatment

**Fig. 5:**
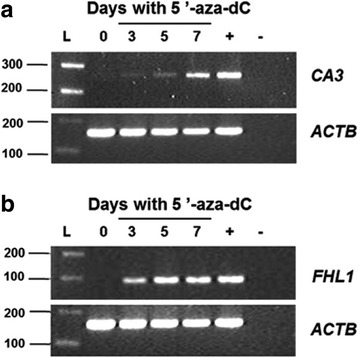
RT-PCR assay for *CA3* and *FHL1* expression analysis after 5'-aza-dC treatment with 1 μM for 7 days. **a** Expression levels of *CA3* in JHU-13 cell line. Note the gradative increase of *CA3* expression from 3 to 7 days; **b** Expression levels of *FHL1* in FaDu cell line. Note that *FHL1* expression was restored at the third day of treatment. *ACTB expression* was used to evaluate the load quantity in each well. + positive control (HCT tumor cell line) and – negative control (without DNA)

In order to evaluate if methylation may contribute to the silencing of gene *CA3* and *FHL1,* the cell lines JHU-13 and FaDu were submitted to 5′-aza-dC treatment. RT-PCR showed that *CA3* downregulation in JHU-13 cell line at day 0 (without 5′-aza-dC treatment) was reverted by 3-day treatment with 1 μM of 5′-aza-dC and the gene expression was gradually recovered until day 7 of treatment (Fig. 5a). *FHL1* gene was not expressed in FaDu cell line, but the 3-day treatment with 1 μM of 5′-aza-dC restored the gene expression (Fig. 5b). Once again, these results suggest that expression of *CA3* and *FHL1* could be regulated by methylation of the promoter region.

## Discussion

A detailed analysis from ORESTES libraries data may be useful on the identification of differentially expressed genes [12, 15, 21, 27]. Based on this concept, this technology was used hereby to identify new candidate genes related to oral carcinogenesis. In silico analysis of ORESTES sequences allowed the identification of 75 differentially expressed genes in the head and neck site, with 64 genes being downregulated. Reis and colleagues [12] conducted a detailed genome mapping analysis of 134,495 ORESTES derived from non-tumor and tumor tissues of the head and neck and thyroid sites. This analysis revealed preferentially expressed genes at the head and neck site as a source of tissue-specific candidate markers for HNSCC.

Twenty-four of the selected genes presented CpG islands in their promoters and had the downregulated expression confirmed by the analysis of head and neck EST public data, reinforcing the idea that ORESTES is a valuable tool to identify differentially expressed genes. According to Strausberg and colleagues [16] the integration of different molecular data sets provides a powerful platform for surveying a wealth of cancer gene expression data in cancer tissues and contributes to the development of new strategies of detection, diagnostic and treatment of this disease. A literature search on the biologic process of the proteins encoded by these 24 genes allowed the selection of 10 downregulated genes to continue in the subsequent analysis. The importance of the experiments in assessing the expression of new candidates is justified by the scarcity of information about genes involved on the molecular events of oral carcinogenesis. RT-PCR analysis showed that eight of these genes presented normal expression in the five HNSCC cell lines evaluated. On the other hand, two genes, *CA3* and *FHL1,* were downregulated in JHU-13 and FaDu cell lines, respectively**.** The evaluation of the expression level of these two genes in OSCC samples by qRT-PCR and in a series of cases and controls from TCGA, demonstrated that *FHL1* and *CA3* were also downregulated in patients´ samples.

Although some differences in the gene expression profile is expected between different subtypes of the head and neck, Chung and colleagues identified 4 different molecular subtypes of HNSCC using patterns of gene expression which were not related to the distinct subsites evaluated (tumors from the oral cavity, oropharynx, hypopharynx and larynx, moreover and normal tissue samples from tonsils). In this study the authors showed that, even though the tumors were from different subsites, the differential gene expression profile did not correlated with tumor subsites but with different molecular and histological features such as EGFR-pathway signature, mesenchymal-enriched subtype, normal epithelium-like subtype, and a subtype with high levels of antioxidant enzymes [28]. In spite of that, we do believe that one potential bias of the gene selection strategy adopted in this study was the use of normal larynx and hypopharynx libraries as a control to compare with oral cancer libraries. Ideally, normal oral cavity tissue should be used in this comparison, however, ORESTES libraries from this subsite were not available in the database. Another putative bias could be the use of cell lines originated from different head and neck subsites during the selection of candidate genes. This step was necessary to identify markers with low expression likely due to epigenetically silencing in tumor cells to be tested in the tumor samples. These strategies may have limited our success in selecting all good candidates for tumor suppressor genes and also allowed the choose of some false positive candidates. During the validation process, we avoid this issue comparing oral cavity tissues from tumor and normal mucosa from healthy donors, however, all data here presented should be further validated with larger dataset containing normal and tumors samples from the oral cavity.

According to previous studies, the most frequent targets for methylation events are the CpG islands situated at gene promoter regions [7]. It is well known that abnormal CpG islands methylation can efficiently repress the transcription of specific genes and act as one of the “hits” in the two-hit Knudson hypothesis of tumor generation [29–31]. Several authors have pointed to a relationship between DNA methylation of tumor suppressor genes such as *p16*, *DAPK* and *MGMT* and the development and progression of head and neck cancers, including oral cancer [32–39]. We therefore reasoned whether aberrant methylation in promoter sites could be the cause of the downregulation observed in the genes selected from the HNSCC ORESTES libraries and started checking for this relationship using in vitro and in silico models. To answer that, we performed the pharmacological unmasking of these cell lines through their treatment with a demethylating agent and observed an upregulation of these genes in the treated cell lines. In silico analysis of TCGA data for normal and OSCC samples showed similar results, with clear-mirrored methylation/expression profiles for both *CA3* and *FHL1* genes. These results reinforced the data, initially obtained from the ORESTES analysis.

The *CA3* gene (carbonic anhydrase III) is a member of a multigene family that encodes carbonic anhydrase isozymes that catalyze the reversible hydration of carbon dioxide to form carbonic acid [40, 41]. Downregulation of this gene was observed in human hepatocelular carcinoma [42] and, according to these authors, the relationship between *CA3* and the response to oxidative stress suggests a role of this gene as a possible mediator of apoptosis or programmed cellular death.

The protein encoded by *FHL1 (Four-and-a-Half LIM-domains 1)* seems to act as a transcriptional factor, and to be associated to focal adherence and intercellular junctions [43]. *FHL1* expression was found downregulated in melanoma and leukemia cell lines [44]. Immunohistochemistry analysis revealed the absence of FHL1 expression in astrocitoma, breast carcinoma, renal carcinoma, hepatocarcinoma, pulmonary adenocarcinoma, prosthatic carcinoma and melanoma tumor samples compared to their corresponding normal tissues [45]. According to these authors, due to its ability in inhibiting specific aspects of tumor cellular growth, *FHL1* could have a tumor suppressor activity [45].

The identification of hypermethylated genes in cancer is extremely important, since silencing confers benefits to the survival of these cells, contributing to a neoplastic phenotype and tumor progression, through the accumulation of genetic and epigenetic *hits* [11]. In the present study, the treatment of JHU-13 and FaDu cell lines with the demethylating agent 5-aza-2′-deoxycytidine was able to restore *CA3* and *FHL1* expression, possibly showing a link between *CA3* and *FHL1* downregulation and aberrant methylation in their promoter sites and a role of methylation in the regulation of these two genes.

Moreover, a recently published study found a significant association of *FHL1* downregulation and its promoter methylation in OSCC cell lines and tumor samples, also suggesting that inactivation of the *FHL1* in OSCCs is through DNA methylation of the promoter region [46].

## Conclusion

In conclusion, our results showed that the downregulation of *CA3* and *FHL1* data observed in silico were validated in HNSCC cell lines and OSCC samples and also suggests that expression of *CA3* and *FHL1* could possibly be regulated by methylation having an important role in the oral carcinogenesis. Moreover, these results warrant further studies for the evaluation of the gene expression and methylation profile of *CA3* and *FHL1* in larger number of samples with clinical and demographic data available to allow the investigation of relevant associations with patient outcome.

## Additional files

Additional file 1: Table S1. List of ORESTES Libraries included in this study. (DOCX 12 kb)

Additional file 2: Table S2. Websites used in the selection of downregulated genes. (DOCX 12 kb)

Additional file 3: Table S3. TCGA sample description. (DOCX 19 kb)

Additional file 4: Table S4. Downregulated genes in head and neck tumors according to the analysis of the ORESTES dataset. (DOCX 15 kb)

Additional file 5: Table S5. List of accession numbers for the ORESTES data used in the study. (XLSX 20500 kb)

## Abbreviations

5-aza-dC: 5-aza-2′-deoxycytidine
BlastN: Basic Local Alignment Search Tool Nucleotide
*CA3*: *Carbonic anhydrase III*
cDNA: Complementary DNA
CpG: 5’-C-phosphate-G-3’
CsCl: Cesium Chloride
Ct: Cycle threshold
DMSO: Dimethyl sulfoxide
dNTP: Deoxynucleotides triphosphates
ESTs: Expressed sequence tags
*FHL1*: *Four-and-a-Half LIM-domains 1*
HNSCC: Head and Neck Squamous Cell Carcinoma
MEM: Minimum Essential Media
MgCl_2_: Magnesium chloride
mRNA: Messenger RNA
NCBI: National Center for Biotechnology Information
ORESTES: Open Reading Expressed Sequence Tags
OSCC: Oral Squamous Cell Carcinoma
qRT-PCR: Quantitative Reverse transcription polymerase chain reaction
RefSeq: Reference Sequences
RPMI: Roswell Park Memorial Institute
RT-PCR: Reverse transcription polymerase chain reaction
SAGE: Serial analysis of gene expression
SAV-CGAP: SAGE Anatomic Viewer - Cancer Genome Anatomy Project
SCC: squamous cell carcinoma
TCGA: The Cancer Genome Atlas
