# Supplementary material for: In vitro and in silico validation of CA3 and FHL1 downregulation in oral cancer
Source: BMC Cancer. 2018 Feb 17;18:193. doi: 10.1186/s12885-018-4077-3 (PMC5816396; doi:10.1186/s12885-018-4077-3)
Supplement: Supplementary file 1 — List of ORESTES Libraries included in this study. (DOCX 12 kb) [file 12885_2018_4077_MOESM1_ESM.docx]

Supplementary Table 1. List of ORESTES Libraries included in this study.

| **Tissue** | Libraries Included | **Total of Libraries** |
| --- | --- | --- |
| **Normal Hypopharynx** | HN0001 to HN0015 | 15 |
| **Normal Larynx** | HN0016 to HN0032 | 16 |
| **Larynx Tumor** | HT0030 to HT0135  HT0400  HT0815 to HT0963  HT0964 to HT1026  HT1128 to HT1172 | 260 |
| **Hipopharynx Tumor** | HT0627 to HT0814 | 545 |
| **Oral Cavity Tumor** | HT0001 to HT0029  HT0136 to HT0399 | 292 |
