# Supplementary material for: In vitro and in silico validation of CA3 and FHL1 downregulation in oral cancer
Source: BMC Cancer. 2018 Feb 17;18:193. doi: 10.1186/s12885-018-4077-3 (PMC5816396; doi:10.1186/s12885-018-4077-3)
Supplement: Supplementary file 2 — Websites used in the selection of downregulated genes. (DOCX 12 kb) [file 12885_2018_4077_MOESM2_ESM.docx]

Supplementary Table 2. Websites used in the selection of downregulated genes.

| **Criteria** | **Website name** | **Website address** |
| --- | --- | --- |
| **Presence of CpG Island in promoter site** | *NCBI Nucleotide*  *UCSC Genome Bioinformatics*  *Methprimer* | http://www.ncbi.nlm.nih.gov/entrez/query.fcgi?db=nucleotide&cmd=search&term=  www.genome.ucsc.edu  http://www.urogene.org/methprimer/index1.html |
| **Downregulation according to ESTs data** | *Virtual Northern SAGE Anatomic Viewer* (SAV) *CGAP* | http://cgap.nci.nih.gov/SAGE/AnatomicViewer |
| **General information (function and biologic process)** | *NCBI GENE*  *NCBI UNIGENE*  *Gene Ontology*  *Gene Cards*  *Source Search*  *USCS Genomic Bioinformatics* | http://www.ncbi.nlm.nih.gov/entrez/query.fcgi?CMD=search&DB=gene  http://www.geneontology.org/  http://bioinfo.weizmann.ac.il/cards/index.shtml  http://genome-www5.stanford.edu/cgi-bin/source/sourceResult  http://genome.ucsc.edu/ |
