# Supplementary material for: In vitro and in silico validation of CA3 and FHL1 downregulation in oral cancer
Source: BMC Cancer. 2018 Feb 17;18:193. doi: 10.1186/s12885-018-4077-3 (PMC5816396; doi:10.1186/s12885-018-4077-3)
Supplement: Supplementary file 3 — TCGA sample description. (DOCX 19 kb) [file 12885_2018_4077_MOESM3_ESM.docx]

Supplementary Table 3. TCGA sample description

| **Sample_ID** | **Sample_Type** | **Anatomic_Site** |
| --- | --- | --- |
| TCGA-CV-6933-11A | Normal | Oral_Tongue |
| TCGA-CV-6934-11A | Normal | Oral_Tongue |
| TCGA-CV-6939-11A | Normal | Oral_Tongue |
| TCGA-CV-6956-11A | Normal | Oral_Tongue |
| TCGA-CV-6959-11A | Normal | Oral_Tongue |
| TCGA-CV-6961-11A | Normal | Oral_Tongue |
| TCGA-CV-7103-11A | Normal | Oral_Tongue |
| TCGA-CV-7238-11A | Normal | Oral_Tongue |
| TCGA-CV-7255-11A | Normal | Oral_Tongue |
| TCGA-CV-6936-11A | Normal | Floor of mouth |
| TCGA-CV-7235-11A | Normal | Floor of mouth |
| TCGA-CV-6938-11A | Normal | Oral_Cavity |
| TCGA-CV-6955-11A | Normal | Oral_Cavity |
| TCGA-CV-7178-11A | Normal | Oral_Cavity |
| TCGA-4P-AA8J-01A | Tumor | Oral_Tongue |
| TCGA-BA-4074-01A | Tumor | Oral_Tongue |
| TCGA-BA-4075-01A | Tumor | Oral_Tongue |
| TCGA-BA-5557-01A | Tumor | Oral_Tongue |
| TCGA-BA-6873-01A | Tumor | Oral_Tongue |
| TCGA-BA-7269-01A | Tumor | Oral_Tongue |
| TCGA-BA-A6DB-01A | Tumor | Oral_Tongue |
| TCGA-BA-A6DE-01A | Tumor | Oral_Tongue |
| TCGA-BA-A6DG-01A | Tumor | Oral_Tongue |
| TCGA-BB-4224-01A | Tumor | Oral_Tongue |
| TCGA-BB-7863-01A | Tumor | Oral_Tongue |
| TCGA-BB-7872-01A | Tumor | Oral_Tongue |
| TCGA-BB-A6UO-01A | Tumor | Oral_Tongue |
| TCGA-C9-A47Z-01A | Tumor | Oral_Tongue |
| TCGA-C9-A480-01A | Tumor | Oral_Tongue |
| TCGA-CN-4725-01A | Tumor | Oral_Tongue |
| TCGA-CN-4733-01A | Tumor | Oral_Tongue |
| TCGA-CN-4736-01A | Tumor | Oral_Tongue |
| TCGA-CN-4737-01A | Tumor | Oral_Tongue |
| TCGA-CN-4742-01A | Tumor | Oral_Tongue |
| TCGA-CN-5367-01A | Tumor | Oral_Tongue |
| TCGA-CN-5370-01A | Tumor | Oral_Tongue |
| TCGA-CN-6017-01A | Tumor | Oral_Tongue |
| TCGA-CN-6019-01A | Tumor | Oral_Tongue |
| TCGA-CN-6024-01A | Tumor | Oral_Tongue |
| TCGA-CN-6996-01A | Tumor | Oral_Tongue |
| TCGA-CN-6998-01A | Tumor | Oral_Tongue |
| TCGA-CQ-5325-01A | Tumor | Oral_Tongue |
| TCGA-CQ-5327-01A | Tumor | Oral_Tongue |
| TCGA-CQ-5329-01A | Tumor | Oral_Tongue |
| TCGA-CQ-5330-01A | Tumor | Oral_Tongue |
| TCGA-CQ-5333-01A | Tumor | Oral_Tongue |
| TCGA-CQ-6219-01A | Tumor | Oral_Tongue |
| TCGA-CQ-6221-01A | Tumor | Oral_Tongue |
| TCGA-CQ-6222-01A | Tumor | Oral_Tongue |
| TCGA-CQ-6224-01A | Tumor | Oral_Tongue |
| TCGA-CQ-6225-01A | Tumor | Oral_Tongue |
| TCGA-CQ-6229-01A | Tumor | Oral_Tongue |
| TCGA-CQ-7065-01A | Tumor | Oral_Tongue |
| TCGA-CQ-7067-01A | Tumor | Oral_Tongue |
| TCGA-CQ-A4CA-01A | Tumor | Oral_Tongue |
| TCGA-CQ-A4CE-01A | Tumor | Oral_Tongue |
| TCGA-CQ-A4CH-01A | Tumor | Oral_Tongue |
| TCGA-CR-6488-01A | Tumor | Oral_Tongue |
| TCGA-CR-6493-01A | Tumor | Oral_Tongue |
| TCGA-CR-7372-01A | Tumor | Oral_Tongue |
| TCGA-CR-7382-01A | Tumor | Oral_Tongue |
| TCGA-CR-7390-01A | Tumor | Oral_Tongue |
| TCGA-CR-7391-01A | Tumor | Oral_Tongue |
| TCGA-CR-7392-01A | Tumor | Oral_Tongue |
| TCGA-CR-7393-01A | Tumor | Oral_Tongue |
| TCGA-CR-7394-01A | Tumor | Oral_Tongue |
| TCGA-CR-7397-01A | Tumor | Oral_Tongue |
| TCGA-CR-7401-01A | Tumor | Oral_Tongue |
| TCGA-CV-5970-01A | Tumor | Oral_Tongue |
| TCGA-CV-5971-01A | Tumor | Oral_Tongue |
| TCGA-CV-5973-01A | Tumor | Oral_Tongue |
| TCGA-CV-5976-01A | Tumor | Oral_Tongue |
| TCGA-CV-5977-01A | Tumor | Oral_Tongue |
| TCGA-CV-5979-01A | Tumor | Oral_Tongue |
| TCGA-CV-6003-01A | Tumor | Oral_Tongue |
| TCGA-CV-6433-01A | Tumor | Oral_Tongue |
| TCGA-CV-6436-01A | Tumor | Oral_Tongue |
| TCGA-CV-6441-01A | Tumor | Oral_Tongue |
| TCGA-CV-6933-01A | Tumor | Oral_Tongue |
| TCGA-CV-6934-01A | Tumor | Oral_Tongue |
| TCGA-CV-6939-01A | Tumor | Oral_Tongue |
| TCGA-CV-6941-01A | Tumor | Oral_Tongue |
| TCGA-CV-6945-01A | Tumor | Oral_Tongue |
| TCGA-CV-6951-01A | Tumor | Oral_Tongue |
| TCGA-CV-6952-01A | Tumor | Oral_Tongue |
| TCGA-CV-6954-01A | Tumor | Oral_Tongue |
| TCGA-CV-6956-01A | Tumor | Oral_Tongue |
| TCGA-CV-6959-01A | Tumor | Oral_Tongue |
| TCGA-CV-6961-01A | Tumor | Oral_Tongue |
| TCGA-CV-7103-01A | Tumor | Oral_Tongue |
| TCGA-CV-7104-01A | Tumor | Oral_Tongue |
| TCGA-CV-7180-01A | Tumor | Oral_Tongue |
| TCGA-CV-7236-01A | Tumor | Oral_Tongue |
| TCGA-CV-7238-01A | Tumor | Oral_Tongue |
| TCGA-CV-7243-01A | Tumor | Oral_Tongue |
| TCGA-CV-7255-01A | Tumor | Oral_Tongue |
| TCGA-CV-7438-01A | Tumor | Oral_Tongue |
| TCGA-CV-7446-01A | Tumor | Oral_Tongue |
| TCGA-CV-A45P-01A | Tumor | Oral_Tongue |
| TCGA-CV-A45R-01A | Tumor | Oral_Tongue |
| TCGA-CV-A45T-01A | Tumor | Oral_Tongue |
| TCGA-CV-A465-01A | Tumor | Oral_Tongue |
| TCGA-CV-A6JO-01B | Tumor | Oral_Tongue |
| TCGA-CV-A6JT-01A | Tumor | Oral_Tongue |
| TCGA-CV-A6JU-01A | Tumor | Oral_Tongue |
| TCGA-CV-A6K0-01B | Tumor | Oral_Tongue |
| TCGA-CX-7085-01A | Tumor | Oral_Tongue |
| TCGA-D6-6515-01A | Tumor | Oral_Tongue |
| TCGA-D6-6823-01A | Tumor | Oral_Tongue |
| TCGA-D6-6825-01A | Tumor | Oral_Tongue |
| TCGA-D6-8569-01A | Tumor | Oral_Tongue |
| TCGA-D6-A4Z9-01A | Tumor | Oral_Tongue |
| TCGA-D6-A4ZB-01A | Tumor | Oral_Tongue |
| TCGA-D6-A6EM-01A | Tumor | Oral_Tongue |
| TCGA-DQ-5624-01A | Tumor | Oral_Tongue |
| TCGA-DQ-5625-01A | Tumor | Oral_Tongue |
| TCGA-DQ-5630-01A | Tumor | Oral_Tongue |
| TCGA-DQ-5631-01A | Tumor | Oral_Tongue |
| TCGA-DQ-7592-01A | Tumor | Oral_Tongue |
| TCGA-F7-A50G-01A | Tumor | Oral_Tongue |
| TCGA-F7-A50J-01A | Tumor | Oral_Tongue |
| TCGA-F7-A61S-01A | Tumor | Oral_Tongue |
| TCGA-F7-A61W-01A | Tumor | Oral_Tongue |
| TCGA-H7-A6C4-01A | Tumor | Oral_Tongue |
| TCGA-HD-7831-01A | Tumor | Oral_Tongue |
| TCGA-HD-8634-01A | Tumor | Oral_Tongue |
| TCGA-HD-8635-01A | Tumor | Oral_Tongue |
| TCGA-HD-A6HZ-01A | Tumor | Oral_Tongue |
| TCGA-IQ-A61E-01A | Tumor | Oral_Tongue |
| TCGA-IQ-A61H-01A | Tumor | Oral_Tongue |
| TCGA-IQ-A61J-01A | Tumor | Oral_Tongue |
| TCGA-IQ-A6SG-01A | Tumor | Oral_Tongue |
| TCGA-IQ-A6SH-01A | Tumor | Oral_Tongue |
| TCGA-KU-A6H8-01A | Tumor | Oral_Tongue |
| TCGA-MT-A51X-01A | Tumor | Oral_Tongue |
| TCGA-MT-A67A-01A | Tumor | Oral_Tongue |
| TCGA-P3-A5QA-01A | Tumor | Oral_Tongue |
| TCGA-QK-A652-01A | Tumor | Oral_Tongue |
| TCGA-QK-AA3K-01A | Tumor | Oral_Tongue |
| TCGA-T2-A6WX-01A | Tumor | Oral_Tongue |
| TCGA-T2-A6WZ-01A | Tumor | Oral_Tongue |
| TCGA-UF-A7JS-01A | Tumor | Oral_Tongue |
| TCGA-UP-A6WW-01A | Tumor | Oral_Tongue |
| TCGA-WA-A7H4-01A | Tumor | Oral_Tongue |
| TCGA-BA-5149-01A | Tumor | Floor of mouth |
| TCGA-BA-5556-01A | Tumor | Floor of mouth |
| TCGA-BA-6872-01A | Tumor | Floor of mouth |
| TCGA-BA-A6D8-01A | Tumor | Floor of mouth |
| TCGA-BA-A6DD-01A | Tumor | Floor of mouth |
| TCGA-BB-8601-01A | Tumor | Floor of mouth |
| TCGA-CN-4730-01A | Tumor | Floor of mouth |
| TCGA-CN-5358-01A | Tumor | Floor of mouth |
| TCGA-CN-5359-01A | Tumor | Floor of mouth |
| TCGA-CN-5364-01A | Tumor | Floor of mouth |
| TCGA-CN-5373-01A | Tumor | Floor of mouth |
| TCGA-CN-6016-01A | Tumor | Floor of mouth |
| TCGA-CN-6995-01A | Tumor | Floor of mouth |
| TCGA-CN-A642-01A | Tumor | Floor of mouth |
| TCGA-CQ-5324-01A | Tumor | Floor of mouth |
| TCGA-CQ-5332-01A | Tumor | Floor of mouth |
| TCGA-CQ-6218-01A | Tumor | Floor of mouth |
| TCGA-CQ-6228-01A | Tumor | Floor of mouth |
| TCGA-CQ-7068-01A | Tumor | Floor of mouth |
| TCGA-CQ-7072-01A | Tumor | Floor of mouth |
| TCGA-CQ-A4C7-01A | Tumor | Floor of mouth |
| TCGA-CQ-A4C9-01A | Tumor | Floor of mouth |
| TCGA-CQ-A4CB-01A | Tumor | Floor of mouth |
| TCGA-CR-6491-01A | Tumor | Floor of mouth |
| TCGA-CV-5436-01A | Tumor | Floor of mouth |
| TCGA-CV-6936-01A | Tumor | Floor of mouth |
| TCGA-CV-6948-01A | Tumor | Floor of mouth |
| TCGA-CV-6953-01A | Tumor | Floor of mouth |
| TCGA-CV-7102-01A | Tumor | Floor of mouth |
| TCGA-CV-7235-01A | Tumor | Floor of mouth |
| TCGA-CV-7407-01A | Tumor | Floor of mouth |
| TCGA-CV-A45X-01A | Tumor | Floor of mouth |
| TCGA-CV-A463-01A | Tumor | Floor of mouth |
| TCGA-CV-A6JD-01A | Tumor | Floor of mouth |
| TCGA-CX-7086-01A | Tumor | Floor of mouth |
| TCGA-CX-7219-01A | Tumor | Floor of mouth |
| TCGA-CX-A4AQ-01A | Tumor | Floor of mouth |
| TCGA-D6-A6EO-01A | Tumor | Floor of mouth |
| TCGA-F7-8489-01A | Tumor | Floor of mouth |
| TCGA-HD-7832-01A | Tumor | Floor of mouth |
| TCGA-HD-7917-01A | Tumor | Floor of mouth |
| TCGA-IQ-A61G-01A | Tumor | Floor of mouth |
| TCGA-KU-A66T-01A | Tumor | Floor of mouth |
| TCGA-MT-A67D-01A | Tumor | Floor of mouth |
| TCGA-MT-A7BN-01A | Tumor | Floor of mouth |
| TCGA-P3-A6T0-01A | Tumor | Floor of mouth |
| TCGA-P3-A6T4-01A | Tumor | Floor of mouth |
| TCGA-P3-A6T7-01A | Tumor | Floor of mouth |
| TCGA-P3-A6T8-01A | Tumor | Floor of mouth |
| TCGA-QK-A6II-01A | Tumor | Floor of mouth |
| TCGA-QK-A6IJ-01A | Tumor | Floor of mouth |
| TCGA-QK-A6VB-01A | Tumor | Floor of mouth |
| TCGA-QK-A8Z7-01A | Tumor | Floor of mouth |
| TCGA-QK-A8Z9-01B | Tumor | Floor of mouth |
| TCGA-T3-A92N-01A | Tumor | Floor of mouth |
| TCGA-UF-A719-01A | Tumor | Floor of mouth |
| TCGA-UF-A71A-01A | Tumor | Floor of mouth |
| TCGA-UF-A71E-01A | Tumor | Floor of mouth |
| TCGA-UF-A7JC-01A | Tumor | Floor of mouth |
| TCGA-UF-A7JO-01A | Tumor | Floor of mouth |
| TCGA-UF-A7JT-01A | Tumor | Floor of mouth |
| TCGA-WA-A7GZ-01A | Tumor | Floor of mouth |
| TCGA-BA-5151-01A | Tumor | Buccal_Mucosa |
| TCGA-CN-4726-01A | Tumor | Buccal_Mucosa |
| TCGA-CN-4731-01A | Tumor | Buccal_Mucosa |
| TCGA-CN-4734-01A | Tumor | Buccal_Mucosa |
| TCGA-CN-A63V-01A | Tumor | Buccal_Mucosa |
| TCGA-CQ-5334-01A | Tumor | Buccal_Mucosa |
| TCGA-CQ-6220-01A | Tumor | Buccal_Mucosa |
| TCGA-CQ-A4C6-01A | Tumor | Buccal_Mucosa |
| TCGA-CQ-A4CG-01A | Tumor | Buccal_Mucosa |
| TCGA-CQ-A4CI-01A | Tumor | Buccal_Mucosa |
| TCGA-CV-6940-01A | Tumor | Buccal_Mucosa |
| TCGA-CV-A464-01A | Tumor | Buccal_Mucosa |
| TCGA-D6-A6EN-01A | Tumor | Buccal_Mucosa |
| TCGA-DQ-7588-01A | Tumor | Buccal_Mucosa |
| TCGA-F7-A624-01A | Tumor | Buccal_Mucosa |
| TCGA-H7-8501-01A | Tumor | Buccal_Mucosa |
| TCGA-HD-A4C1-01A | Tumor | Buccal_Mucosa |
| TCGA-IQ-7631-01A | Tumor | Buccal_Mucosa |
| TCGA-P3-A6T2-01A | Tumor | Buccal_Mucosa |
| TCGA-QK-A6IG-01A | Tumor | Buccal_Mucosa |
| TCGA-UF-A7JA-01A | Tumor | Buccal_Mucosa |
| TCGA-UF-A7JD-01A | Tumor | Buccal_Mucosa |
| TCGA-BA-5558-01A | Tumor | Hard_Palate |
| TCGA-CN-5369-01A | Tumor | Hard_Palate |
| TCGA-CQ-5331-01A | Tumor | Hard_Palate |
| TCGA-CQ-7063-01A | Tumor | Hard_Palate |
| TCGA-CR-6492-01A | Tumor | Hard_Palate |
| TCGA-CV-5442-01A | Tumor | Hard_Palate |
| TCGA-QK-A64Z-01A | Tumor | Hard_Palate |
| TCGA-BA-5152-01A | Tumor | Alveolar_Ridge |
| TCGA-BA-A6DJ-01A | Tumor | Alveolar_Ridge |
| TCGA-CN-4741-01A | Tumor | Alveolar_Ridge |
| TCGA-CN-6011-01A | Tumor | Alveolar_Ridge |
| TCGA-CN-6013-01A | Tumor | Alveolar_Ridge |
| TCGA-CN-A49A-01A | Tumor | Alveolar_Ridge |
| TCGA-CQ-5323-01A | Tumor | Alveolar_Ridge |
| TCGA-CQ-5326-01A | Tumor | Alveolar_Ridge |
| TCGA-CQ-6223-01A | Tumor | Alveolar_Ridge |
| TCGA-CQ-7069-01A | Tumor | Alveolar_Ridge |
| TCGA-CV-A45O-01A | Tumor | Alveolar_Ridge |
| TCGA-IQ-7632-01A | Tumor | Alveolar_Ridge |
| TCGA-P3-A5QF-01A | Tumor | Alveolar_Ridge |
| TCGA-P3-A6T5-01A | Tumor | Alveolar_Ridge |
| TCGA-P3-A6T6-01A | Tumor | Alveolar_Ridge |
| TCGA-QK-A6IH-01A | Tumor | Alveolar_Ridge |
| TCGA-T2-A6X2-01A | Tumor | Alveolar_Ridge |
| TCGA-UF-A71B-01A | Tumor | Alveolar_Ridge |
| TCGA-BB-A5HU-01A | Tumor | Oral_Cavity |
| TCGA-BB-A5HZ-01A | Tumor | Oral_Cavity |
| TCGA-CN-4728-01A | Tumor | Oral_Cavity |
| TCGA-CN-4729-01A | Tumor | Oral_Cavity |
| TCGA-CN-4740-01A | Tumor | Oral_Cavity |
| TCGA-CN-6018-01A | Tumor | Oral_Cavity |
| TCGA-CN-6020-01A | Tumor | Oral_Cavity |
| TCGA-CN-6994-01A | Tumor | Oral_Cavity |
| TCGA-CQ-6227-01A | Tumor | Oral_Cavity |
| TCGA-CQ-7071-01A | Tumor | Oral_Cavity |
| TCGA-CQ-A4CD-01A | Tumor | Oral_Cavity |
| TCGA-CR-6471-01A | Tumor | Oral_Cavity |
| TCGA-CR-6484-01A | Tumor | Oral_Cavity |
| TCGA-CR-7365-01A | Tumor | Oral_Cavity |
| TCGA-CR-7367-01A | Tumor | Oral_Cavity |
| TCGA-CR-7368-01A | Tumor | Oral_Cavity |
| TCGA-CR-7369-01A | Tumor | Oral_Cavity |
| TCGA-CR-7373-01A | Tumor | Oral_Cavity |
| TCGA-CR-7376-01A | Tumor | Oral_Cavity |
| TCGA-CR-7377-01A | Tumor | Oral_Cavity |
| TCGA-CR-7379-01A | Tumor | Oral_Cavity |
| TCGA-CR-7380-01A | Tumor | Oral_Cavity |
| TCGA-CR-7386-01A | Tumor | Oral_Cavity |
| TCGA-CR-7395-01A | Tumor | Oral_Cavity |
| TCGA-CV-5966-01A | Tumor | Oral_Cavity |
| TCGA-CV-6937-01A | Tumor | Oral_Cavity |
| TCGA-CV-6938-01A | Tumor | Oral_Cavity |
| TCGA-CV-6942-01A | Tumor | Oral_Cavity |
| TCGA-CV-6955-01A | Tumor | Oral_Cavity |
| TCGA-CV-6960-01A | Tumor | Oral_Cavity |
| TCGA-CV-7090-01A | Tumor | Oral_Cavity |
| TCGA-CV-7091-01A | Tumor | Oral_Cavity |
| TCGA-CV-7095-01A | Tumor | Oral_Cavity |
| TCGA-CV-7097-01A | Tumor | Oral_Cavity |
| TCGA-CV-7099-01A | Tumor | Oral_Cavity |
| TCGA-CV-7100-01A | Tumor | Oral_Cavity |
| TCGA-CV-7178-01A | Tumor | Oral_Cavity |
| TCGA-CV-7183-01A | Tumor | Oral_Cavity |
| TCGA-CV-7252-01A | Tumor | Oral_Cavity |
| TCGA-CV-7253-01A | Tumor | Oral_Cavity |
| TCGA-CV-7254-01A | Tumor | Oral_Cavity |
| TCGA-CV-7263-01A | Tumor | Oral_Cavity |
| TCGA-CV-7409-01A | Tumor | Oral_Cavity |
| TCGA-CV-7411-01A | Tumor | Oral_Cavity |
| TCGA-CV-7413-01A | Tumor | Oral_Cavity |
| TCGA-CV-7414-01A | Tumor | Oral_Cavity |
| TCGA-CV-7416-01A | Tumor | Oral_Cavity |
| TCGA-CV-7423-01A | Tumor | Oral_Cavity |
| TCGA-CV-7425-01A | Tumor | Oral_Cavity |
| TCGA-CV-7427-01A | Tumor | Oral_Cavity |
| TCGA-CV-7428-01A | Tumor | Oral_Cavity |
| TCGA-CV-7429-01A | Tumor | Oral_Cavity |
| TCGA-CV-7432-01A | Tumor | Oral_Cavity |
| TCGA-CV-7434-01A | Tumor | Oral_Cavity |
| TCGA-CV-7435-01A | Tumor | Oral_Cavity |
| TCGA-CV-7568-01A | Tumor | Oral_Cavity |
| TCGA-CV-A45Q-01A | Tumor | Oral_Cavity |
| TCGA-CV-A45U-01A | Tumor | Oral_Cavity |
| TCGA-CV-A45V-01A | Tumor | Oral_Cavity |
| TCGA-CV-A6JE-01A | Tumor | Oral_Cavity |
| TCGA-CV-A6JN-01A | Tumor | Oral_Cavity |
| TCGA-CV-A6JY-01A | Tumor | Oral_Cavity |
| TCGA-CV-A6JZ-01A | Tumor | Oral_Cavity |
| TCGA-CV-A6K2-01A | Tumor | Oral_Cavity |
| TCGA-CX-7082-01A | Tumor | Oral_Cavity |
| TCGA-H7-7774-01A | Tumor | Oral_Cavity |
| TCGA-H7-8502-01A | Tumor | Oral_Cavity |
| TCGA-HD-A633-01A | Tumor | Oral_Cavity |
| TCGA-HD-A6I0-01A | Tumor | Oral_Cavity |
| TCGA-HL-7533-01A | Tumor | Oral_Cavity |
| TCGA-MT-A67F-01A | Tumor | Oral_Cavity |
| TCGA-P3-A6T3-01A | Tumor | Oral_Cavity |
| TCGA-RS-A6TO-01A | Tumor | Oral_Cavity |
