# Supplementary material for: In vitro and in silico validation of CA3 and FHL1 downregulation in oral cancer
Source: BMC Cancer. 2018 Feb 17;18:193. doi: 10.1186/s12885-018-4077-3 (PMC5816396; doi:10.1186/s12885-018-4077-3)
Supplement: Supplementary file 4 — Downregulated genes in head and neck tumors according to the analysis of the ORESTES dataset. (DOCX 15 kb) [file 12885_2018_4077_MOESM4_ESM.docx]

Supplementary Table 4. Downregulated genes in head and neck tumors according to the analysis of the ORESTES dataset

| **Gene ID** | **Simbol** | **Definition** |
| --- | --- | --- |
| NM_001100 | *ACTA1* | *Actin, alpha 1, skeletal muscle* |
| NM_001155 | *ANXA6* | *Annexin A6* |
| NM_198098 | *AQP1* | *Aquaporin 1 (Colton blood group)* |
| NM_001660 | *ARF4* | *ADP-ribosylation factor 4* |
| NM_016607 | *ARMCX3* | *Armadillo repeat containing, X-linked 3* |
| NM_182810 | *ATF4* | *Activating transcription factor 4 (tax-responsive enhancer element B67)* |
| NM_173201 | *ATP2A1* | *ATPase, Ca++ transporting, cardiac muscle, fast twitch 1* |
| NM_178540 | *C1QTNF9* | *C1q and tumor necrosis factor related protein 9* |
| NM_033197 | *C20orf114* | *Chromosome 20 open reading frame 114* |
| NM_001010905 | *C6orf58* | *Chromosome 6 open reading frame 58* |
| NM_032307 | *C9orf64* | *Chromosome 9 open reading frame 64* |
| NM_005181 | *CA3* | *Carbonic anhydrase III* |
| NM_006835 | *CCNI* | *Cyclin I* |
| NM_001001547 | *CD36* | *CD36 molecule (thrombospondin receptor* |
| NM_021914 | *CFL2* | *Cofilin 2* |
| NM_001824 | *CKM* | *Creatine kinase, muscle* |
| NM_018941 | *CLN8* | *Ceroid-lipofuscinosis, neuronal 8* |
| NM_133507 | *DCN* | *Decorin (DCN),* |
| NM_004396 | *DDX5* | *DEAD (Asp-Glu-Ala-Asp) box polypeptide 5* |
| NM_004417 | *DUSP1* | *Dual specificity phosphatase 1* |
| NM_012179 | *FBXO7* | *F-box protein 7* |
| NM_001449 | *FHL1* | *Four and a half LIM domains 1* |
| NM_007085 | *FSTL1* | *Follistatin-like 1* |
| NM_002046 | *GAPDH* | *Glyceraldehyde-3-phosphate dehydrogenase* |
| NM_006496 | *GNAI3* | *Guanine nucleotide binding protein (G protein), alpha inhibiting activity polypeptide 3* |
| NM_002084 | *GPX3* | *Glutathione peroxidase 3* |
| NM_002128 | *HMGB1* | *High-mobility group box 1* |
| NM_006353 | *HMGN4* | *High mobility group nucleosomal binding domain 4* |
| NM_002227 | *JAK1* | *Janus kinase 1* |
| NM_014686 | *KIAA0355* | *KIAA0355* |
| XM_113947 | *KIAA0565* | *KIAA0565 gene product* |
| NM_005356 | *LCK* | *Lymphocyte-specific protein tyrosine kinase* |
| NM_007078.2 | *LDB3* | *LIM domain binding 3* |
| XM_498824 | *LOC149157* | *Hypothetical protein LOC149157* |
| XM_496332 | *LOC440552* | *Similar to OK/SW-CL.16* |
| XM_497531 | *LOC441786* | *Similar to NADH2 dehydrogenase (ubiquinone) (EC 1.6.5.3) chain 1 - western lowland gorilla mitochondrion* |
| NM_002465 | *MYBPC1* | *Myosin binding protein C* |
| NM_005963 | *MYH1* | *Myosin, heavy polypeptide 1* |
| NM_017534 | *MYH2* | *Myosin, heavy chain 2* |
| NM_000257 | *MYH7* | *Myosin, heavy polypeptide 7* |
| NM_079420 | *MYL1* | *Myosin, light polypeptide 1* |
| NM_000432 | *MYL2* | *Myosin, light polypeptide 2* |
| NM_032578 | *MYPN* | *Myopalladin* |
| NM_004543 | *NEB* | *Nebulin* |
| NM_003204 | *NFE2L1* | *Nuclear factor (erythroid-derived 2)-like 1* |
| NM_006175 | *NRAP* | *Nebulin-related anchoring protein* |
| NM_000917 | *P4HA1* | *Procollagen-proline, 2-oxoglutarate 4-dioxygenase (proline 4-hydroxylase), alpha polypeptide I* |
| NM_138316 | *PANK1* | *Pantothenate kinase 1* |
| NM_022817 | *PER2* | *Period homolog 2* |
| NM_145753 | *PHLDB2* | *Pleckstrin homology-like domain, family B, member 2* |
| NM_021033 | *RAP2A* | *RAP2A, member of RAS oncogene family* |
| NM_018993 | *RIN2* | *Ras and Rab interactor 2* |
| NM_000540 | *RYR1* | *Ryanodine receptor 1* |
| NM_005620 | *S100A11* | *S100 calcium binding protein A11* |
| NM_016103 | *SAR1B* | *SAR1 gene homolog B (S. cerevisiae)* |
| NM_001636 | *SLC25A6* | *Solute carrier family 25 (mitochondrial carrier;adenine nucleotide translocator), member 6* |
| NM_006282 | *STK4* | *Serine/threonine kinase 4* |
| NM_003254 | *TIMP1* | *TIMP metallopeptidase inhibitor 1* |
| NM_003262 | *TLOC1* | *Translocation protein 1* |
| NM_003282 | *TNNI2* | *Troponin I type 2* |
| NM_152263 | *TPM3* | *Ttropomyosin 3 (TPM3)* |
| NM_133378 | *TTN* | *Titin (TTN),* |
| NM_021009 | *UBC* | *Ubiquitin C* |
| NM_025160 | *WDR26* | *WD repeat domain 26* |
